# Using Virtual Reality in the Inference-Based Treatment of Compulsive Hoarding

**DOI:** 10.3389/fpubh.2016.00149

**Published:** 2016-07-19

**Authors:** Marie-Eve St-Pierre-Delorme, Kieron O’Connor

**Affiliations:** ^1^Institut universitaire en santé mentale de Montréal, Montreal, QC, Canada

**Keywords:** compulsive hoarding, virtual reality, treatment, cognitive therapy, inference-based therapy

## Abstract

The present study evaluated the efficacy of adding a virtual reality (VR) component to the treatment of compulsive hoarding (CH), following inference-based therapy (IBT). Participants were randomly assigned to either an experimental or a control condition. Seven participants received the experimental and seven received the control condition. Five sessions of 1 h were administered weekly. A significant difference indicated that the level of clutter in the bedroom tended to diminish more in the experimental group as compared to the control group *F*(2,24) = 2.28, *p* = 0.10. In addition, the results demonstrated that both groups were immersed and present in the environment. The results on posttreatment measures of CH (*Saving Inventory revised, Saving Cognition Inventory and Clutter Image Rating scale*) demonstrate the efficacy of IBT in terms of symptom reduction. Overall, these results suggest that the creation of a virtual environment may be effective in the treatment of CH by helping the compulsive hoarders take action over their clutter.

## Introduction

Though classified in DSM-V as a distinct disorder, compulsive hoarding (CH) was long considered a subtype of OCD. Also new in DSM-V is that OCD is no longer considered an anxiety disorder. CH and OCD are now considered separate disorders, part of the Obsessive Compulsive and Related Disorders category. Regardless, the experience of intense anxiety when required to get rid of personal objects and difficulty in taking action are part of the diagnostic criteria for CH (1). The majority of studies investigating CH and OCD were conducted when CH was still considered as anxiety disorder. Virtual reality (VR) is a rapidly growing area of technology that is being used more and more as an adjunct to treatment for various mental health problems. VR facilitates exposure in that almost any context or situation can be simulated (2). Studies have found that VR is effective for the treatment of panic disorder (3), social phobia (4), obsessive–compulsive disorder (OCD) (5, 6), post-traumatic stress disorder (7, 8), specific phobia (9, 10), generalized anxiety disorder (11), and eating disorders (12). To our knowledge, no study has yet investigated the treatment of CH with VR.

A meta-analysis that evaluated the use of VR for anxiety disorders found that the effect sizes were quite large for the 21 included studies, with the average effect size being 0.95 (13). Another meta-analysis that exclusively looked at the use of VR for anxiety disorders (*n* = 13) corroborates the results of the other meta-analysis and reports an average effect size of 1.11 for VR, as compared to a control condition (14). When considering the effect size for VR in comparison to an *in vivo* condition, the authors describe an effect size of 0.35 in favor of VR. Furthermore, in several controlled trials, VR was found to be as effective as *in vivo* exposure (15).

A few studies have also examined environments known as “non-immersive,” which are generated using a standard computer. These studies found that the use of non-immersive environments can allow for a state of presence in the environment, elicit an emotional response equivalent to an immersive environment, and can lead to significant amelioration of clinical symptoms (4, 16). It is, therefore, possible to infer that the creation of an environment simulating CH conditions can provoke anxiety as well as encourage sorting items and uncluttering in the therapeutic process.

### Compulsive Hoarding

Compulsive hoarding is characterized by a number of behaviors, such as cluttered rooms and difficulty or refusal to get rid of unnecessary objects. These symptoms cause an important level of distress, which interferes with everyday life (1). Individuals with CH also tend to be indecisive, perfectionistic, and disorganized. They also often procrastinate and have an urgent need to save and acquire a diverse array of objects (17). It has also been observed that hoarders take significantly more time to sort, create more piles, and experience more anxiety than non-psychiatric controls, but this is only true when dealing with their own personal objects (18).

The efficacy of existing treatments for CH is limited (19). In addition, studies cite certain issues with using exposure, as hoarders tend to drop out of treatment at this step, and many others are reluctant to let the therapist enter their homes (19). As they have difficulty in making decisions, it is not easy for hoarders to take action once it is time for them to organize their environment or remove clutter. They also have difficulty in completing homework, and their lack of motivation is often an issue in the therapeutic process (20, 21). That said, VR represents an avenue to explore in the treatment of CH, as it circumvents the aforementioned difficulties that usually represent important obstacles in therapy.

### Context of the Problem

As VR allows for precise control over what is presented, it is possible to create environments tailored to the individual, based on the needs of the client (6). Also, VR can be helpful for CH as it is controlled, predictable, and reliable (22). In addition to these advantages, VR can also be administered in the clinical context (without having to leave the office) and costs less (15). This tool can also be useful for individuals, like CH, who have difficulties with visualizing everyday scenes when using mental imagery techniques (23). Furthermore, the clutter in the homes of the hoarders is very visual, and VR is a technology that primarily uses this aspect of the sensory system. All of these reasons support VR as a viable alternative to at-home visits to facilitate action taking in hoarders.

The objectives of the present article are to evaluate whether VR is an efficacious component in helping hoarders to take action toward reducing clutter and to validate the efficacy of a group version of inference-based therapy (IBT) validated in an individual format (Blais et al. forthcoming)^1^.

The hypotheses:

1- There will be a statistically significant difference between the experimental and control conditions on measures of clutter, such as the Clutter Image Rating scale.
2- The participants in the experimental condition will experience clinically greater levels of anxiety than participants in the control condition during the sessions with immersion in the VR environment.
3- The non-immersive virtual environment will elicit a state of presence and immersion in all of the participants.
4- IBT will lead to clinically and statistically significant improvement in symptoms of (a) CH, (b) anxiety, and (c) depression.

## Materials and Methods

### Participants

Participants were recruited using advertisements posted in universities, hospitals, CLSC’s, and community organizations in the region of Montréal, as well as Fernand Seguin research centre’s website. Twenty-five participants were evaluated in the context of this project. Of these, nine were excluded following the initial evaluation, and two dropped out over the course of treatment, one in each group. Of the 14 participants who took part in the project, 2 were males and 12 were females. Demographic data are reported in Table 1. Inclusion criteria were as follows: (a) a primary diagnosis of OCD with CH as described in DSM-IV-TR as well as the criteria proposed for DSM-V; (b) stable medication for at 12 weeks; (c) accept to keep medication stable throughout participation in the study; (d) no evidence of current suicidal ideation; (e) no evidence of current alcohol or drug abuse; (f) no evidence or diagnosis of schizophrenia past or present, bipolar disorder, or organic mental disorder; and (g) accept to not receive any other treatment for CH during the course of the study. Excluded participants were referred to the most appropriate resources, given their situation. Comorbid symptoms, such as obsessive–compulsive disorder traits and other subtype of obsessive–compulsive disorder, were observed for seven participants. Participants with mild comorbidity were included, three participants had mild depressive symptoms, six had other subtypes of OCD symptoms and four had obsessive–compulsive personality traits.

**Table 1:** **Demographic data**.

	Experimental		Control	
Variables	Mean	SD	Mean	SD
Age	50.71	7.70	50.00	11.74

	**Frequency**	**%**	**Frequency**	**%**

Education level				
Elementary	0	0	0	0
High school	1	14.29	3	42.86
CEGEP	0	0	1	14.29
University	6	85.71	3	42.86
Individual income				
10,000–19,999$	2	28.57	1	14.29
20,000–29,999$	2	28.57	3	42.86
30,000–39,999$	2	28.57	1	14.29
40,000–59,999$	0	0	1	14.29
60,000$+	1	14.29	1	14.29
Civil status				
Single	3	42.86	3	42.86
Married or in a relationship	2	28.57	3	42.86
Divorced or separated	1	14.29	0	2.86
Widowed	1	14.29	1	14.29
Occupation				
Full time	4	57.14	2	28.57
Part time	1	14.29	1	14.29
Jobless	2	28.57	4	57.14
Medication				
Antidepressant	3	42.86	3	42.86
No medication	4	57.14	4	57.14

### Equipment

The environments were generated by a PC with the following specifications: PC Pentium 4®, 1.98 GHz 3.48 GB of RAM, with 256 MB of video memory. The environments were projected on a 21″-monitor. The *neuroVR* 2.0 platform (www.neurovr.org) was used to create the environments. A digital camera was used to take photographs of the CH’s home environments and *Corel Paint Shop Pro Photo X2* software was used to treat the images. This procedure was validated at the Université du Québec en Outaouais (UQO) in a sample of six participants (24).

### Virtual Reality

#### Experimental Environment

All of the virtual environments were created using the neuroVR 2.0 platform (www.neurovr.org), which is freely accessible online. In the experimental condition, objects that belong to the hoarders were inserted into the apartment environment created using this platform. In the control condition, images depicting objects were selected from the internet and inserted into the apartment environment. To create the experimental virtual environments, approximately 30 photos taken by participants of their homes were selected. The objects in these images were cut out using *Corel Paint Shop Pro Photo X2* and inserted in the apartment environment. The objective was to recreate the participant’s apartment, mainly their living room, kitchen, and bedroom. The objects were placed in piles as represented in their pictures. The objects that were eventually selected to be used in VR procedure were cut out individually so that the participant could select them.

#### Control Environment

**Figure 1:**
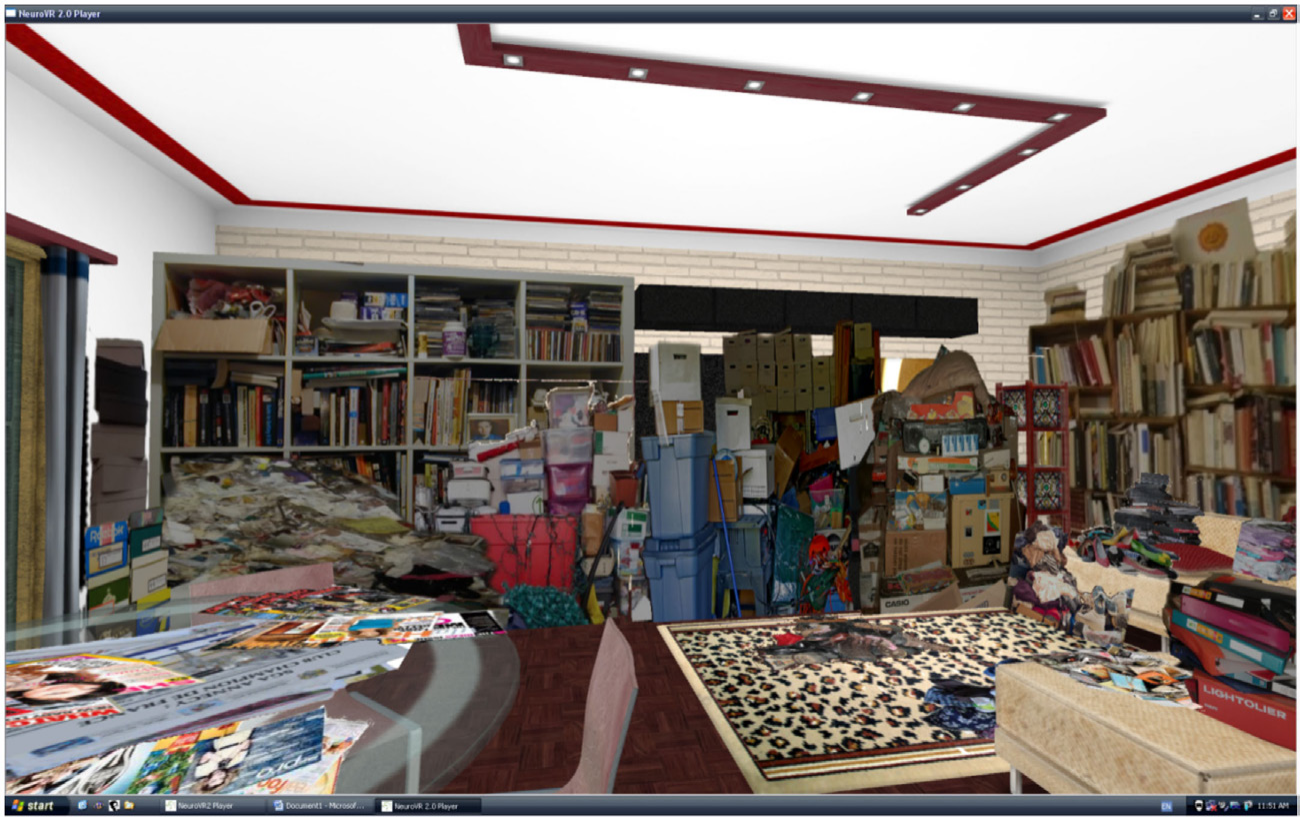
**Living room of the control environment**.

An active control condition (see Figure 1) was chosen as opposed to a passive control condition. This active type of condition was preferred because it allows for an equivalence of parameters, such as the number of sessions and contact with a therapist. For participants in the control group, the virtual environment was created in the same way as for the participants in the experimental group. The only difference was that the objects did not belong to the participants. Common household objects were selected like shoes, books, magazines, frames, etc. The selected objects came from the internet, the homes of the therapists, and the research center.

### Clinical Evaluations

A battery of questionnaires and semi-structured interviews were administered to all participants who took part in the project. The purpose of these measures was to obtain information regarding the presenting problem of the individual, to establish the presence of depression and anxiety, and to measure the state the participant was in, following each VR task. The self-report measures were administered pretreatment (IBT), post-IBT treatment, and post-VR. The clinical interviews were administered by an evaluator trained in the administration of CH measures. The evaluation lasted for 4 h.

The *Structured Clinical Interview for DSM-IV* for Axis I disorders [SCID-I; French version; Ref. (25)] was used to establish a differential diagnosis for Axis I disorders according to the diagnostic criteria of the DSM-IV-TR. These measures have very good psychometric properties (26).

The *Yale–Brown Obsessive Compulsive Scale* [YBOCS; Ref. (27); French translation; Ref. (28)] was used in the clinical evaluation of obsessive–compulsive symptoms and their severity. The YBOCS can also be used to evaluate overt and covert neutralization behaviors, separately. Studies have found support for the validity and reliability of these subscales (ICC = 0.01–0.94, *r*_s_ = 0.90) (29).

The *Overvalued Ideas Scale* (OVIS) (30) is an 11-item semi-structured interview evaluating overvalued ideas across several dimensions (e.g., efficacy of compulsions, degree of belief held by others, etc.). This measure is often used to measure the degree of introspection of individuals suffering from different obsessional disorders, such as OCD. The OVIS has satisfactory internal consistency (α = 0.88), test–retest reliability (*r* = 0.86), and inter-rater reliability (*r* = 0.88).

The *Evaluation of Primary Inferences Scale* (EPIS) (31) was developed to measure the strength of the primary obsessional doubt. This measure is complementary to and more specific than the OVIS, with regards to primary doubts. With the help of a psychologist, the participant must identify their primary inferences regarding their obsessions and determine for each their level of conviction (%) in terms of the probability that this belief is real in the “here and now.” For a hoarder, the primary inference is often formulated as: maybe I can repair this object or maybe I can save some money.

The *Evaluation of Secondary Inferences Scale* (EPIS) (31). With the help of a psychologist, the participant must identify their secondary inferences (anticipated consequences of the primary inferences) and answer for each the following question: “please evaluate to what extent (%) inferences described here are realistic if you do not perform your compulsions.”

#### Symptom Measures

Three measures of hoarding were used to evaluate the severity of hoarding based on the formal definition of the problem. The first questionnaire, the *Saving Inventory-Revised* (SIR) (32), is comprised of 23 items scored on a scale from 0 to 4. The subscales are (a) compulsive acquisition, (b) difficulty discarding objects, and (c) clutter in the home environment. These subscales have been found to have good reliability.

The *Saving Cognitions Inventory* (SCI) (33) is a questionnaire comprised of 24 items measuring beliefs related to CH symptoms. There are four main subscales: (a) emotional attachment, (b) preoccupation regarding memory, (c) need for control, and (d) responsibility regarding possessions. The internal consistency of these subscales is good and varies between 0.86 and 0.95.

The *Clutter Image Rating* (CIR) (34) is a series of nine images that correspond to different degrees of severity of clutter. The rooms depicted are the kitchen, living room, and bedroom. This measure has good internal consistency (α = 0.84) and intercorrelations between 0.56 and 0.71. A score of 4 or more reflects the presence of CH symptoms, and each room receives its own score. There is no total score.

The *Beck Depression Inventory* (BDI-II) (35) is a 21-item self-report questionnaire, which aims to evaluate affective, cognitive, motivational, and physiological symptoms of depression during the last 2 weeks. Like the original English version, the French translation has good psychometric qualities, such as internal consistency (α = 0.92–0.93) and test–retest reliability (*r* = 0.93).

The *Beck Anxiety Inventory* (BAI) (36) contains 21 items designed to evaluate the intensity of anxiety symptoms during the last week. Like the original English version, the French translation has acceptable internal consistency (α = 0.84) and test–retest reliability (*r* = 0.63) (37).

The *Inferential Confusion Questionnaire – Expanded Version* (ICQ-EV) (38) is comprised of 30 items measuring inferential confusion, a construct referring to how an individual accords a certain degree of probability to imaginary possibilities (39). This measure has excellent internal consistency (α = 0.97) and test–retest reliability (*r* = 0.90).

##### VR Measures

The Canadian adaptation of the *Immersive Tendencies Questionnaire* (ITQ) (40) is comprised of 18 items divided into four subscales: (a) focus, (b) implication, (c) emotions, and (d) game. A study by Witmer and Singer (40) demonstrated good psychometric qualities, and the French translation validation study reported a Cronbach’s alpha of 0.78 (41).

The French Canadian adaptation of the *Presence Questionnaire* (PQ-F) (40) consists of 24 items and seven subscales: (a) realism, (b) possibility to take action, (c) quality of the interface, (d) possibility to examine, (e) self-evaluation of performance, (f) auditory, and (g) tactile. The authors report good internal consistency. It is also worth noting that the last two subscales were not used in the present study, as they are optional and no auditory or tactile element was used.

The French Canadian translation of the Simulator Sickness Questionnaire (SSQ-F) (42) was also administered. The two main subscales are nausea and oculomotor problems. A study evaluating this French-language version reports an excellent Cronbach’s alpha of 0.87 (43).

Finally, the therapist asked the participants to rate their level of anxiety and discomfort during the VR tasks. For example, when the client had to discard a virtual object, the therapist asked the client to rate their level of anxiety and discomfort on a scale from 0 to 10 and took note of the rating. This allows for measure of the difficulty of the task and also to verify if the VR elicited the expected emotions.

### Procedure

Fourteen participants with a diagnosis of CH took part in this project. The participants were randomly assigned to one of two of the following conditions: active control condition and experimental condition.

#### Treatment Protocol

##### Inference-Based Therapy

All of the participants received IBT administered by psychologists trained in this approach. The treatment protocol used was developed by O’Connor et al. (31) and adapted for CH clients (44). Participants received 24 group format sessions, each lasting for 1½ h. The sessions were audio recorded and verified by an independent person involved with the project to ensure the integrity of the steps of the treatment was respected. The 10 steps of IBT adapted for CH are the following: (i) distinguish normal from CH obsessional doubt; (ii) establish the logic of the CH doubt; (iii) the CH doubt is 100% imaginary; (iv) how CH becomes a “lived” experience; (v) crossing the border of reality; (vi) and (vii) reasoning devices in CH; (viii) establish the selective nature of the imaginary doubt; (ix) vulnerability of these in the CH stories; and (x) awareness of reality and tolerating the void. Treatment was the same in both conditions.

##### Virtual Reality

After receiving IBT, five sessions of 1 h were given to participants in both conditions. These sessions were administered by psychologists trained in the use of neuroVR 2.0 and the established treatment protocol. These sessions were also audio recorded and verified by an independent person involved with the project to ensure the integrity of the steps of the VR sessions was respected. In addition, the participants had no contact with one another between VR sessions. The first session allowed participants in the experimental condition to familiarize themselves with the virtual environment and to change elements of the environment to make them resemble, as much as possible, their actual environment at home. The second session was aimed at helping participants begin to sort through their homes by establishing a plan of action based on the elements present in the virtual environment. In the last three sessions, participants took action virtually by disposing of objects already selected based on the degree of subjective distress they reported. Once an object was selected, the participants were supposed to put it in a virtual, 3D garbage can. They were then asked to re-evaluate their degree of anxiety and discomfort on a scale of 0 to 10. At the beginning of each session, the therapist evaluated if some objects were sorted or taken out from home by the participant. The last session was also used to discuss relapse prevention and to establish a plan of action for the coming months. The client was asked to identify at-risk situations for relapse and to write a plan of action with the therapist as a way to ensure the client had all of the tools that may be needed in the future. Finally, measures of presence and VR sickness were completed at the end of each VR session.

For control participants, the steps of treatment were the same. The only difference was that the objects selected when sorting and discarding did not belong to them. Their level of anxiety and discomfort on a scale from 0 to 10 was still evaluated to determine the presence of any emotions toward these objects. With regards to the task completed at home, participants were asked to discard one object, without specifying which one. The point of this exercise was to see if they would dispose of an object that was similar to the one discarded in the virtual environment.

### Statistical Analysis

Normality was verified for all variables and the analyses were conducted by taking into consideration whether the assumptions were met or not. Analysis was conducted on completers. Repeated measures ANOVAs and *T*-test were conducted on all normal variables. Non-parametric analyses were conducted when variables did not meet the assumptions of normality or had a small sample size. The variables for which non-parametric analyses were conducted are the following: the subscale “control” of the SCIR and the “acquisition” and clutter subscales of the SIR. The non-parametric analyses of these variables will be presented following the presentation of the parametric analyses. Additionally, a significance level of 0.10 was chosen because of the small sample size. Even though we used a cut-off point of 0.10, we nonetheless corrected for multiple comparison analysis.

## Results

### Pretreatment Analyses

Univariate (age) and chi-square (sex, civil status, level of education, medication, and revenue) analyses did not find any significant differences between the groups (*p* > 0.05). Also, there were no significant differences between the groups at pretreatment on clinical measures.

### Pre-VR and Post-VR Analyses

#### Main Measures

Repeated measures ANOVAs were conducted on the main measure of clutter, the CIR. With regards to the images of the bedroom, a significant interaction was observed *F*(2,24) = 2.28, *p* = 0.10. It is possible to qualify this interaction because of the significance level. Contrasts indicated a linear interaction between the two groups for the pre-VR and post-VR *F*(1,12) = 7.80, *p* < 0.001. This interaction demonstrates that the participants’ results for the series of images of the bedroom in the experimental group had a tendency to decrease over time as compared to the control group. There were no interactions observed for the other rooms depicted in the CIR, the kitchen *F*(2,24) = 0.16, *p* = 0.85 and the living room *F*(2,24) = 0.90, *p* = 0.42.

Paired sample *t*-tests were conducted in order to determine if there was a difference between the mean level of anxiety before and after action was taken in the two groups. A significant difference was observed for the experimental group *t*(6) = 17.67, *p* < 0.001 as well as for the control group *t*(6) = 8.00, *p* < 0.001. An independent *t*-test was conducted, and a significant difference was found between the groups before action was taken in VR *t*(12) = 3.36, *p* < 0.05 and after the VR task *t*(12) = 3.35, *p* < 0.05.

#### Secondary Measures

A repeated measures ANOVA demonstrated a main effect of time *F*(2,24) = 5.32, *p* < 0.05, but no interaction effect, for the “emotional” subscale of the SCIR *F*(2,24) = 0.97, *p* = 0.39. There was also a main effect of time for the “responsibility” subscale of the SCIR *F*(2,24) = 4.20, *p* < 0.05 and the “memory” subscale *F*(2,24) = 7.76, *p* < 0.05, but no significant interaction effects between groups (*p* > 0.05). A Greenhouse–Geisser correction was applied to these variables. Finally, a main effect of time was observed for the SCIR total score *F*(2,24) = 9.46, *p* < 0.001, but there were no interaction effects.

As previously mentioned, a main effect of time from pre-IBT to post-RV was found for the SIR total score and its three subscales. A repeated measures ANOVA did not find an interaction between the groups for the “discarding objects” subscale *F*(1,12) = 2.38, *p* = 0.15 or for the SIR total score *F*(1,12) = 1.68, *p* = 0.22. As the “clutter” and “acquisition” subscales did not meet the assumptions of normality, non-parametric analyses were conducted. There is no test equivalent to a 2 × 2 repeated measures analysis. As such, a composite score between post-RV and pre-IBT was calculated and a Mann–Whitney *U* test was conducted in order to determine if there was a significant difference between groups. No difference was observed for the “clutter” subscale for the experimental group (Md = 0.00, *n* = 7) and the control group (Md = 2.00, *n* = 7), *U* = 20.00, *z* = −0.58, *p* = 0.56, *r* = 0.14. Also, there was no difference for the “acquisition” subscale for the experimental group (Md = 12.28, *n* = 7) and control group (Md = 3.00, *n* = 7), *U* = 14.00, *z* = −1.35, *p* = 0.18, *r* = 0.36.

#### Other Clinical Questionnaires

A repeated measures ANOVA found a main effect of time for the total score on the YBOCS between pre-IBT, post-IBT, and post-RV *F*(2,24) = 3.56, *p* < 0.05, and no interaction effect *F*(2,24) = 0.16 *p* = 0.85. A main effect of time was also observed for the “obsessions” subscale *F*(2,24) = 4.19, *p* < 0.05 but not for the “compulsions” subscale *F*(2,24) = 1.13, *p* = 0.31. No interaction was observed between the groups for either subscale. A repeated measures ANOVA found no main effect *F*(2,24) = 2.39, *p* = 0.11 or interaction *F*(2,24) = 0.24, *p* = 0.24 for the OVIS. There was, however, a linear trend across time for both groups. Essentially, the total score demonstrated a tendency to decrease for the two groups. The results do not demonstrate a main effect for the BDI *F*(2,22) = 0.02, *p* = 0.98 or the BAI *F*(2,22) = 0.22, *p* = 0.80. There was also no interaction observed for the BDI *F*(2,22) = 1.81, *p* = 0.19. There was, however, an interaction found for the BAI *F*(2,22) = 3.17, *p* < 0.10. The BAI scores for the control group had a tendency to decrease over time, while that of the experimental group increased from pre-IBT to post-IBT, and decreased at post-VR. A Mann–Whitney *U* test did not find any difference between the groups on the ICQ-EV at post-VR: experimental group (Md = 85, *n* = 6) and control group (Md = 82, *n* = 7), *U* = 19.5, *z* = −0.22, *p* = 0.83, *r* = 0.06. See Table 2 for a synthesis of these results. Table 2 also shows differences in pre and post-IBT as well as interactions between the two groups post-VR.

**Table 2:** **Mean and SD of the clinical measures**.

	Experimental				Control			
Measures	N	Pre	Post	Post-VR	N	Pre	Post	Post-VR
YBOCS								
Total	7	20.86 (4.74)	17.57 (4.16)*	18.43 (5.32)	7	21.71 (3.04)	18.86 (3.48)*	20.57 (5.00)
Obsessions	7	9.86 (2.34)	8.71 (2.36)*	8.71 (2.87)	7	11.43 (2.07)	8.86 (2.79)*	9.71 (3.59)
Compulsions	7	8.38 (2.83)	8.86 (1.95)*	9.71 (2.69)	7	10.29 (1.80)	10.00 (1.41)*	10.86 (1.68)
OVIS	7	61.43 (7.91)	50.71 (9.46)	51.57 (7.50)	7	61.28 (10.14)	61.43 (11.87)	58.29 (9.23)
BAI	7	8.83 (3.06)	12.83 (5.64)	11.83 (7.46)*	7	14.16 (14.90)	11.50 (13.09)	10.50 (11.88)*
BDI	7	22.29 (13.15)	21.86 (12.47)	23.67 (15.07)	7	20.57 (14.02)	20.57 (14.02)	17.00 (11.89)
SIR total	7	71.71 (10.90)	–	67.43 (17.23)	7	67.42 (7.96)	–	58.85 (7.36)
Clutter	7	27.20 (6.57)	–	28.00 (9.05)	7	28.86 (3.67)	–	27.57 (5.44)
Discarding/saving	7	22.87 (3.12)	–	15.91 (5.70)	7	20.86 (4.38)	–	16.86 (3.98)
Acquisition	7	21.80 (3.35)	–	16.00 (5.20)	7	17.71 (5.56)	–	14.43 (4.79)
SCIR total	7	109.69 (20.76)	92.79 (22.89)**	97.70 (10.34)	7	111.98 (24.73)	88.93 (22.20)**	90.14 (17.18)
Emotional	7	44.08 (11.30)	37.54 (14.13)*	40.68 (4.45)	7	45.08 (14.88)	36.68 (12.60)*	34.86 (10.71)
Responsibility	7	24.71 (7.01)	19.53 (4.04)*	19.49 (6.64)	7	25.28 (6.99)	19.38 (6.90)*	21.43 (4.28)
Memory	7	22.55 (7.48)	19.52 (6.55)**	20.41 (5.86)	7	23.11 (6.67)	17.52 (6.50)**	17.29 (5.53)
Control	7	18.33 (2.42)	16.21 (3.74)*	17.12 (1.58)	7	18.40 (1.34)	15.35 (1.70)*	16.57 (2.57)
ICQ-EV	7	78.71 (26.87)	83.67 (24.06)	85.50 (23.80)	7	78.43 (40.88)	61.29 (34.32)	86.14 (52.35)
CIR room	7	4.66 (2.13)	3.58 (1.52)	3.41 (1.90)*	7	4.57 (2.44)	4.72 (1.97)	4.28 (2.21)*
Kitchen	7	4.59 (2.29)	4.08 (2.13)	4.12 (2.41)	7	3.71 (1.11)	3.37 (1.25)	3.57 (1.51)
Living room	7	4.18 (1.57)	3.87 (2.19)	4.32 (1.89)	7	4.29 (2.43)	4.29 (1.60)	4.14 (1.72)

*p < 0.10.

**p < 0.001.

*YBOCS, Yale–Brown obsessive compulsive scale; OVIS, overvalued ideation scale; BAI, Beck anxiety inventory; BDI, Beck depression inventory; SIR, saving inventory revised; SCIR, saving cognition inventory revised; ICQ-EV, inferential confusion questionnaire extended version; CIR, Clutter Image Rating*.

#### VR Measures

Independent *t*-tests found no significant difference between groups on any of the subscales of the presence questionnaire. The scores demonstrated a high degree of presence for both groups according to the French-language norms of the questionnaire; experimental group M = 90.96, SD = 3.07; control group M = 86.98, SD = 20.94. The SD is much larger for the control group than for the experimental group. An independent *t*-test did not find any difference between the two groups for the Simulator Sickness Questionnaire. Furthermore, each group obtained scores that were below the average reported in the norms for the questionnaire (experimental: M = 4.6, SD = 6.10; control: M = 7.27, SD = 2.95).

### Comparison with a Non-Concurrent Control Group

We also compared the results of this study with a non-concurrent data collected from another group. Five participants were recruited in the same way as the participants in the present study’s sample and participated in the first therapy group given at the Fernand Seguin research center, before the virtual environments were created. They received the same treatment protocol (IBT), and it was the same therapist who animated the sessions.

Measures were taken at pretreatment, posttreatment, and at 6 months following completion of the treatment. The results demonstrate no clinical or statistical difference on measures of clutter (CIR) from pre-IBT to post-IBT; bedroom: *z* = 0.00, *p* = 1.0; kitchen: *z* = −1.00, *p* = 0.32; living room: *z* = 0.00, *p* = 1.00. This was also the case for measures from post-IBT to 6-month follow-up: bedroom: *z* = 0.00, *p* = 1.0; kitchen: *z* = −1.41, *p* = 0.20; living room: *z* = 0.00, *p* = 1.00. The results for the three rooms decreased.

With regards to the secondary measures, scores on the YBOCS decreased significantly from pre-IBT to post-IBT *z* = −1.6, *p* < 0.10, with a large effect size (*r* = 0.57). The median score decreased from pre-IBT (Md = 21.50) to post-IBT (Md = 17.00). The “obsessions” subscale decreased significantly *z* = −1.83, *p* < 0.10, with a large effect size (*r* = 0.65). The median score decreased from pre-IBT (Md = 11.00) to post-IBT (Md = 8.50). No change, however, was observed for the “compulsions” subscale *z* = −0.38, *p* = 0.71. There was no significant difference for the total YBOCS score from post-IBT to 6-month follow-up *z* = −0.00, *p* = 1.00.

There was no significant difference from pre-IBT to post-IBT on the OVIS: *z* = −0.37, *p* = 0.72 and BDI: *z* = −0.92, *p* = 0.36. There was a significant difference on the ICQ-EV *z* = −1.60, *p* < 0.10, with a large effect size (*r* = 0.60). The median score decreased from pre-IBT (Md = 76.00) to post-IBT (Md = 65.50).

A significant difference on the BAI was also observed *z* = −1.83, *p* = 0.06, with a large effect size (*r* = 0.65). The median score decreased from pre-IBT (Md = 16.50) to post-IBT (Md = 7.00). There was no significant difference on any of the questionnaires from post-IBT to 6-month follow-up.

With regards to the secondary CH measures, no significant decrease on the SIR total score and the “clutter” and “discarding objects” subscales was observed between pre-IBT and 6-month follow-up. A significant difference was observed on the “acquisition” subscale *z* = −1.63, *p* < 0.10, with a large effect size (*r* = 0.62). The median score decreased from pre-IBT (Md = 2.00) to 6-month follow-up (Md = 0.00). No measures were taken at post-IBT in this sample, as they were in the present study.

With regards to the SCIR, no significant difference was observed from pre-IBT to post-IBT for the total score or for its three subscales: “emotional,” “responsibility,” and “memory.” A significant difference was observed for the “control” subscale from pre-IBT to post-IBT *z* = −1.63, *p* < 0.10, with a large effect size (*r* = 0.62). The median score decreased from pre-IBT (Md = 2.00) to post-IBT (Md = 0.00). No significant difference was observed from post-IBT to the 6-month follow-up for the SCIR and its subscales.

### Pretreatment IBT and Posttreatment IBT Analyses

#### CH Questionnaires

Paired sample *t*-tests were conducted to measure change on measure of CH from pre-IBT to post-IBT. Unfortunately, the SIR was not completed at post-IBT because of an omission in the questionnaire package given to participants. As such, the only measures available for the SIR are at pre-IBT and post-VR. A significant difference was observed from pre-IBT to post-VR for the SIR total score *t*(13) = 4.79, *p* < 0.001 and the “discarding objects” subscale *t*(13) = 5.44, *p* < 0.001. A Wilcoxon test of signed-rank showed a statistically significant decrease on the “acquisition” subscale from pre-IBT to post-VR *z* = −2.64, *p* < 0.005, with a large effect size (*r* = 0.50). The median score on the “acquisition” subscale decreased between pre-IBT (Md = 21) and post-VR (Md = 15). No significant difference was observed for the “clutter” subscale between pre-IBT and post-VR *z* = −1.03, *p* = 0.31.

A significant difference was found for the SCIR total score from pre-IBT to post-IBT, *t*(13) = 4.59, *p* < 0.001, and for its three subscales: “emotional” *t*(13) = 2.82, *p* < 0.05; “responsibility” *t*(13) = 3.07, *p* < 0.05; and “memory” *t*(13) = 6.07, *p* < 0.001.

A Wilcoxon signed-rank test demonstrated a statistically significant decrease for the subscale “control” of the SCIR from pre-IBT to post-IBT *z* = −2.80, *p* < 0.005, with a large effect size (*r* = 0.53). The median score decreased between pre-IBT and post-TBI (Md = 15.46).

No significant difference was found between the different images on the CIR between pre-IBT and post-IBT (bedroom: *t*(13) = 1.14, *p* = 0.27; kitchen: *t*(13) = 1.30, *p* = 2.22; living room: *t*(13) = 0.54, *p* = 0.60).

A repeated measures ANOVA was conducted comparing the two groups (experimental and control) for all measures of CH from pre-IBT to post-IBT and no significant interaction was observed between the two groups (*p* > 0.05).

#### Other Clinical Measures

Paired sample *t*-tests were conducted and demonstrated a significant decrease in YBOCS total score from pre-IBT to post-IBT *t*(13) = 3.21, *p* < 0.05, and for the “obsessions” subscale *t*(13) = 2.77, *p* < 0.05. Also, a significant difference was observed for the “compulsions” subscale *t*(13) = 3.21, *p* < 0.06. There was no significant difference on the OVIS *t*(13) = 1.37, *p* = 0.20, BDI *t*(13) = 1.00, *p* = 0.34, or BAI *t*(13) = −0.09, *p* = 0.93. To better understand these results, a repeated measures ANOVA was conducted and the results demonstrated that for the BAI, an interaction effect was present between the two groups *F*(1,12) = 8.64, *p* < 0.05. The scores of the experimental group increased, whereas those of the control group decreased. As the assumption of sphericity was not met, the Greenhouse–Geisser correction was applied. As the ICQ-EV was not normally distributed and also not transformable, a Wilcoxon signed-ranked test was conducted. There was no significant difference from pre-IBT to post-IBT for the ICQ-EV *z* = −0.72, *p* = 0.47.

## Discussion

As expected, the results on measures of VR demonstrated a good state of presence during the experimentation, good immersion, and very little VR sickness. These results confirm the hypothesis that non-immersive virtual environments allow for the creation of a feeling of presence and immersion, which can contribute to the eliciting of emotions, when using the virtual environment.

It was observed that both groups experienced anxiety during the VR sessions, a finding that is contrary to the initial hypothesis that stipulated that the control group would not experience significant anxiety. A significant difference, however, was found between the groups. Specifically, the participants in the experimental group experienced significantly more anxiety than the control group during the action taking task completed in the virtual environment. Following the VR task, the control group was significantly less anxious than the experimental group. This may be explained by the fact that the virtual environment in the experimental condition used participants’ personal objects, whereas the virtual environments in the control condition did not. For this reason, the feeling of attachment toward the objects was not the same for participants in each condition.

Following VR sessions, there was an interaction between the groups on the main measure of clutter, that is, for the images of the bedroom. The level of clutter in the experimental group showed a tendency to decrease, as compared to a tendency to increase in the control group. This may signify that taking action in VR allowed participants in the experimental group to discard significantly more objects in their bedroom. It does not seem that action taken in the control group allowed participants to discard objects in the same room, as their scores actually increased. It should be noted, however, that for participants in the experimental group, the majority of selected pictures were of their bedroom.

Following VR, there was also a decrease over time in cognitions related to the CH symptoms “responsibility,” “emotional,” and “memory” as well as for the total score. The SIR total score as well as the “acquisition” and “discarding objects” subscales also decreased over time, as well as the “obsessions” subscale of the YBOCS. Overvalued ideas also evinced a tendency to decrease over the three time points, pre-IBT, post-IBT, and post-VR. Finally, a significant interaction was observed between the groups on a measure of anxiety. The results of the experimental group increased slightly at post-IBT and decreased at post-VR, whereas anxiety in the control group consistently decreased over time. This may be explained by the fact that individuals in the experimental group had to submit pictures of their home so that their virtual environment could be created. This may have caused anxiety as many of the participants in this group reported worries about what was going to happen in the VR sessions.

Finally, the comparison of the experimental group with the control group and non-concurrent comparison group suggest that the VR condition is an accessible and interesting tool that may help the participant take action at home. The results also demonstrate that IBT is a promising approach in the treatment of CH as it was possible to observe improvements in CH symptoms in the participants. It may also be possible to use VR as a preventative measure to impede the development of compulsive behavior (45).

After receiving IBT, participants in all three groups evinced a significant improvement in their CH-related cognitions. The same can be said for the measures of obsessions and compulsions, as these scores significantly decreased as well for both groups included in the present study. No differences were observed, however, on measures of depression or inferential processes. Also, no differences were observed on the measure of clutter (CIR). These results are in line with other studies that have reported that hoarders have difficulty in taking action when it comes to their clutter. With regards to the measure of depression, the participants expressed that they would have appreciated additional sessions. The majority of them reported feeling discouraged regarding the clutter of their homes and did not see any progress in this respect. With regards to overvalued ideas, even though the scores on the OVIS did not significantly decrease, the clinical scales demonstrated a decrease of 40% in the primary doubt. This pattern of results may be attributable to the fact that the OVIS considers only one overvalued idea, whereas the clinical scales are more exhaustive, taking into account all of the overvalued ideas reported by the participant.

The limitations of the present study are mainly the small sample size and the absence of a passive control group. Also, it would have been preferable to have additional time points, such as at 3- and 1-year follow-up. Following the clinical evaluations post-VR, some participants indicated they had experienced difficulties during the IBT and the VR. As such, it would have been of interest to have included qualitative measures and analyses. For example, participants reported problems, such as bankruptcy, sickness or death of a loved one, eviction, and loss of employment. These elements were also corroborated by the participants’ psychologists. The participants as well as the psychologists identified these events as obstacles to progress in treatment. It is, therefore, important to consider these life events as they can have a considerable impact on the success of treatment.

Other elements should also be considered for future research. Indeed, it would be interesting to compare the experimental condition to a passive control condition or a wait list. Though the results were compared to a non-concurrent control group that did not receive VR, these participants were not randomly assigned to this condition. Also, as many studies include home visits, it would be of interest to have an active control condition comparing VR to home visits. In addition, it may be that five sessions of VR is insufficient to observe significant results on CH measures. As the home environments are extremely cluttered, they require a lot of work to achieve satisfactory results.

In conclusion, it seems that non-immersive VR is accessible and elicits a state of presence and immersion in participants suffering from CH. VR also elicits emotions during sorting tasks and when virtually discarding objects. Personalization of the virtual environment seems to help hoarders clean out their environment, as was the case in the bedroom in the context of this study. Only two participants dropped out of treatment, which is very little compared to the majority of studies conducted with this population. It may be that VR is less overwhelming. Finally, the participants reported that virtually sorting and discarding objects helped them to take action and experience less distress and anxiety at home. They were also generally satisfied with the IBT and VR.

## Author Contributions

The present article was part of M-E St-P-D’s thesis. Dr. KO was supervising the entire research.

## Conflict of Interest Statement

The authors declare that the research was conducted in the absence of any commercial or financial relationships that could be construed as a potential conflict of interest.

## Footnotes

^1^Blais MT, Bodryzlova Y, Aardema F, O’Connor K. Open trial of inference-based therapy in the treatment of compulsive hoarding. *J Behav Addict* (Forthcoming).
